# Disruptive Effects of Colorful vs. Non-colorful Play Area on Structured Play—A Pilot Study with Preschoolers

**DOI:** 10.3389/fpsyg.2016.01661

**Published:** 2016-10-28

**Authors:** Keren Stern-Ellran, Sigal Zilcha-Mano, Rachel Sebba, Nava Levit Binnun

**Affiliations:** ^1^Faculty of Architecture and Town Planning, Technion – Israel Institute of TechnologyHaifa, Israel; ^2^Department of Psychology, University of HaifaHaifa, Israel; ^3^Baruch Ivcher School of Psychology, Sagol Center for Brain and Mind, Interdisciplinary Center HerzliyaHerzliya, Israel

**Keywords:** sensory enrichment, preschoolers, colorfulness, visual, attention, environment, educational psychology, cognitive development

## Abstract

To contribute to young children's development, sensory enrichment is often provided via colorful play areas. However, little is known about the effects of colorful environments on children while they engage in age-appropriate tasks and games. Studies in adults suggest that aspects of color can distract attention and impair performance, and children are known to have less developed attentional and executive abilities than adults. Preliminary studies conducted in children aged 5–8 suggest that the colorfulness of both distal (e.g., wall decorations) and proximal (e.g., the surface of the desktop) environments can have a disruptive effect on children's performance. The present research seeks to extend the previous studies to an even younger age group and focus on proximal colorfulness. With a sample of 15 pre-schoolers (3–4 years old) we examined whether a colorful play surface compared to a non-colorful (white) play surface would affect engagement in developmentally appropriate structured play. Our pilot findings suggest that a colorful play surface interfered with preschoolers' structured play, inducing more behaviors indicating disruption in task execution compared with a non-colorful play surface. The implications of the current study for practice and further research are discussed.

## Introduction

Childhood learning environments, especially in kindergarten and elementary school, are often rich with colorful educational materials and other visual displays (Fisher et al., 2014). Extensive colorfulness is used in children's play areas not only in books and toys but also in playground facilities, furniture, carpets, and wall decorations (Sebba, 2005). Media based stimulation (such as television and computer screens) are nowadays an integral part of many play areas (Christakis et al., 2012).

The underlying motivation for these colorful and sensory-rich environments is to elicit the positive, and often crucial, developmental effects that have been found to occur when sensory enrichment is provided during early stages of development (Lewis and Maurer, 2009; Sale et al., 2009; Baroncelli et al., 2010; Clemenson et al., 2015). However, there may be a threshold beyond which children's surroundings become *excessively* stimulating and disrupting (Godwin and Fisher, 2011; Thompson and Raisor, 2013; Choi et al., 2014; Fisher et al., 2014) and instead of serving as a beneficial enriched environment, they can become a “cacophony of imagery” (Tarr, 2004, p. 1) or a distracting “visual bombardment” (Bullard, 2016, p. 110).

In studies on adult populations, several visual elements that are often used to design sensory enriched environments were found to distract attention and impair performance (e.g., Rodrigues and Pandeirada, 2015). For example, colorfulness—one of the easiest ways to create a sensory enriched environment and a major feature of children's surroundings—may at times become excessively stimulating for adults. Specifically, contrast in brightness (e.g., a light blue object adjacent to a dark blue object, but also two adjacent saturated colors such as a saturated yellow object adjacent to a saturated green object) was found to be a very potent distractor to various tasks (Turatto and Galfano, 2000, 2001; Franconeri and Simons, 2003; Lambert et al., 2003; Franconeri et al., 2005; Fuller et al., 2009). Similar, though milder, effects were also demonstrated for contrast in hue (i.e., wave length; Turatto and Galfano, 2001; Lambert et al., 2003; Lu and Zhou, 2005).

It is difficult to generalize these findings in adults to children in a straightforward manner. On the one hand, visual stimulation may serve as beneficial enrichment in children, much more than in adults. On the other hand, children's attentional abilities and executive functions are still developing (Sarid and Breznitz, 1997; Lopez et al., 2005; Gaspelin et al., 2015) suggesting that children's visual environments may not always be advantageous (Choi et al., 2014). Indeed, while reflexive (automatic) attention is observed shortly after birth (Plude et al., 1994), other aspects of attention, such as the ability to suppress irrelevant information and sustain attention, have a rather long course of development (e.g., Ruff and Capozzoli, 2003; Rueda et al., 2004; Kannass and Colombo, 2007; Bartgis et al., 2008; Fisher et al., 2013; Gaspelin et al., 2015). It was found that toddlers are more prone to attention hijacking by distracting stimuli than are older children, and even at the age of 10 years, children are more susceptible to interference than are adults (Goldberg et al., 2001; Durston et al., 2002). Notably, the ability to sustain attention appropriately to objects, events, and tasks is considered important in many kinds of learning and performance (Ruff and Lawson, 1990). Thus, it is essential to gain a deeper understanding of the competitive environmental stimulations that children encounter during their everyday exploration (Kannas et al., 2006).

Despite this growing understanding of children's distractibility, only a few studies have directly investigated the effects of children's visual environments on their performance. The few available studies suggest a potential adverse effect of colorful backgrounds on children's performance. Ksantini-Hovev and Sebba (2005) showed a negative influence of a colorful desktop background on 8-year-olds' performance in regular school tasks. Eight-year-old children completed sets of equivalent tests in their regular school class, in two different environmental conditions: Desktops were either covered with a white or with a colorful board. In all tests, it took the children less time to complete the assignments on the white background. Moreover, in tests that required written answers (as opposed to multiple choice or math), achievements were significantly higher with the white background. Godwin and Fisher (2011) tested younger children, about 5 years of age, while manipulating the distal visual environment of a laboratory, modifying it to look like a classroom. In the decorated-classroom condition, the walls were decorated with science posters, maps and the children's art work. In the sparse-classroom condition, all materials irrelevant to ongoing instruction were removed. Lessons consisted of a short read-aloud, during which illustrations in a book were shown to the children. Then, the children were given a pencil and paper visual task pertaining to the completed lesson. They found that children spent more time off-task in the decorated-classroom condition. A follow-up study that used a similar design (Fisher et al., 2014) extended these results and demonstrated an adverse effect of the surroundings' colorfulness on children's achievement. Specifically, children achieved lower scores in the decorated-classroom condition compared to the sparse-classroom condition.

Although, still preliminary, these studies suggest that both the distal (e.g., wall decorations) and proximal (e.g., colorfulness of the desktop) colorfulness of children's environments can affect children's performance. While Ksantini-Hovev and Sebba (2005) studied older children and focused on proximal effects, Godwin and Fisher (2011) and Fisher et al. (2014) studied younger children and focused on distal colorfulness. The present research seeks to extend the previous studies to an even younger age group and focus on proximal colorfulness. This type of study conducted with younger children seems especially warranted for the following reasons: (1) Paradoxically, younger children, who are more prone to distraction, are usually surrounded by more colorful and decorated environments (Fisher et al., 2014), manifested in wall decorations, as well as in the colorfulness of furniture, carpets, and surrounding toys. (2) Moreover, in many cases the colorfulness is obtained by using combinations of saturated colors, resulting in sharp contrasts of hue and brightness (Sebba, 2005).

In this pilot study, young preschoolers (aged three to four) engaged in developmentally appropriate structured play on a non-colorful (white) and on a colorful surface, while their behavior was monitored. The colorful surface was characterized by highly saturated colors, creating sharp hue, and brightness contrasts, and served as an operational representation of a child's natural proximal play area (e.g., playing on a colorful carpet). Based on the previous findings with older children, we hypothesized that, relative to a non-colorful play surface, a highly colorful one would interfere with young children's structured play and would induce more behaviors indicating disruption in task execution.

## Methods

### Experiment design

In order to examine the effect of proximal colorfulness on preschoolers' play, a within-subject design was employed, with a manipulation of the surface colorfulness, such that children aged three to four played in two environments, with a colorful (C) and a non-colorful (NC) background, one after the other in a randomized order. In each condition, each child played three games (puzzle assembly, Lego reconstruction, and picture card Lotto, see details below). Before the test condition phase, a pretest phase was conducted (for full details of the pretest phase see Supplementary Material). In this phase, the experimenter played with the each child with the games to be used later in the test phase. The purpose of the pretest phase was two-fold. First, to adjust the individual level of the games for each child, as it is known that at these early ages, individual differences can be extensive (Korkman et al., 2010). Second, the pretest phase enabled accommodation of the children with the experiment, minimizing any effects of unfamiliarity with the games used in the test phase, the experiment room or the experimenter (K.S.E). At the end of the pretest phase, all children had played all the three games to be encountered in the test-phase at least four times, and their game level was adjusted to accommodate for developmental differences (see Supplementary Material for details on the way game levels were determined).

### Participants

The pilot study was conducted at a private preschool in one of Israel's central cities and carried out in accordance to the guidance and approval of the local institutional ethics committee. The parents of all children aged three to four (*N* = 28 months) were invited to a lecture on children's cognitive development in toddlerhood. At the end of the lecture, parents were given a brief update about the study and were asked to approve their child's participation and sign a consent form. Of the 25 children whose parents signed the consent form, three children were unwilling to cooperate with the experimenter. Of the remaining 22 children who participated in the pretest, only 18 continued to the test phase. This was due to failure in understanding the tasks or in completing them without intense interaction with the experimenter (*N* = 3) or lack of sufficient cooperation (*N* = 1). Of the 18 children who participated in the test phase, two were excluded due to scheduling limitations. An additional child displayed constant movement and physical agitation throughout the sessions and was also excluded from the analysis. The remainder of children in the test phase (*N* = 15) who were included in the analysis were between 38 and 52 months old (*M* = 44.1 months, *SD* = 4.37 months) at the time of the first data collection. These children did not wear glasses and were assumed to have normal vision. During the sessions themselves, one child had to go to the restroom, two children were interested in engaging with the experimenter rather than in the task, and one child complained of a stomach ache. This resulted in full data sets (data for both C and NC conditions) from 14 children in the puzzle task, 15 for the Lego task and 12 for the Lotto task.

### Experimental conditions and tools

#### Surface background

**Figure 1:**
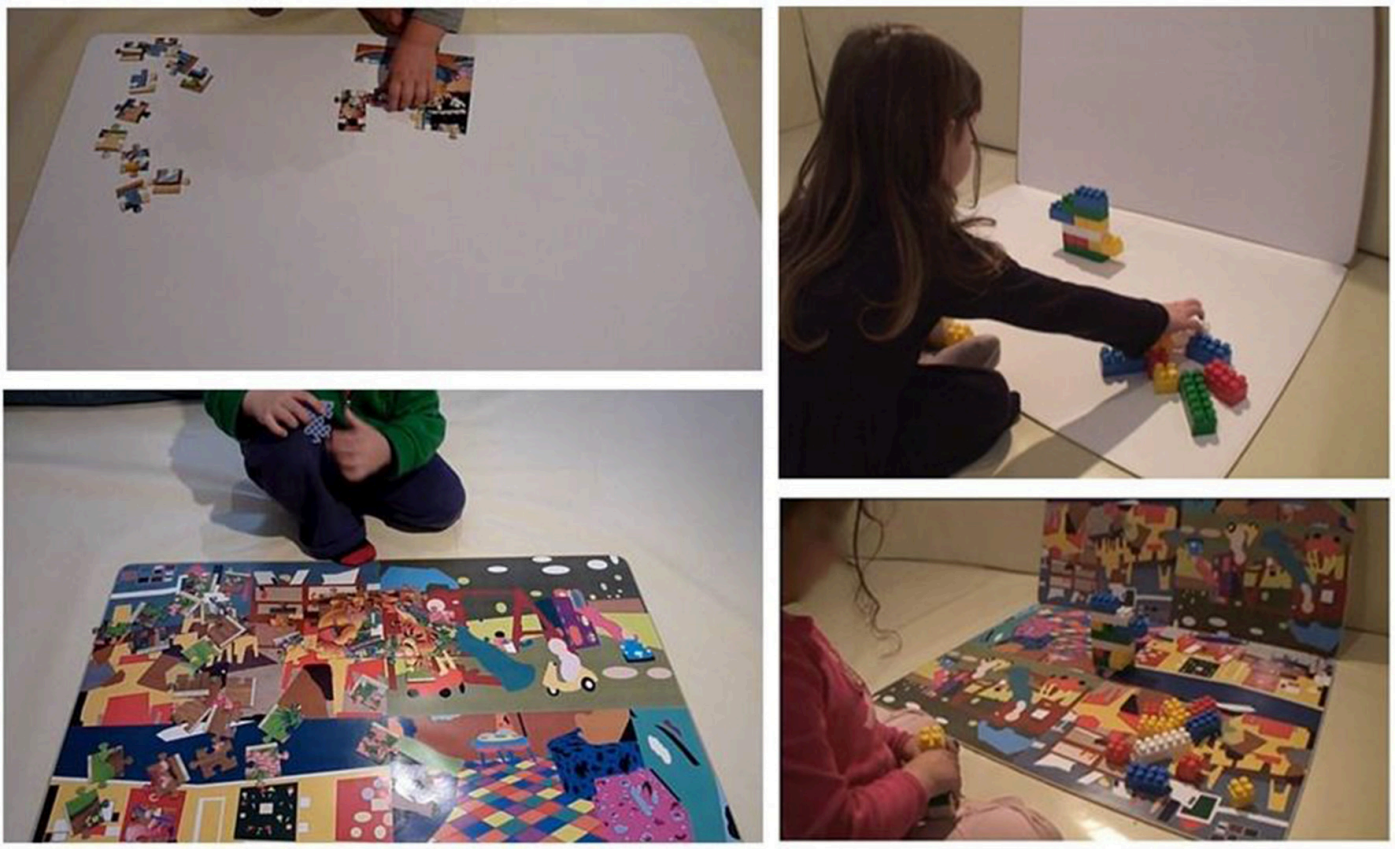
**Non-colorful and colorful conditions**. The upper pictures depict the non-colorful (NC) background and the lower pictures depict the colorful (C) background. Here the child is playing with the puzzle (pictures on the right) and the Lego (pictures on the left) tasks.

In the non-colorful (NC) condition all surfaces were covered with white paper (see Figure 1, upper panel). In the colorful (C) condition, surfaces were covered with colorful paper, which had sharp hue and brightness contrasts, created by computer processing of images of children's play environments (see Figure 1, lower panel). For more details regarding how these colorful surfaces were created and their ecological validity, see Supplementary Material.

The surface (NC or C) on which the children played was a 59.5 × 83.5 cm plywood board (subtending a visual angle of ~40°). A similar plywood surface with the same dimensions and color was placed vertically, behind the horizontal one, so that it was in front of the child (see Figure 1). Each condition was conducted on separate days, but on the same day of the week and approximately at the same time of day. The two test runs were between 1 and 2 weeks apart. To control for order and gender effects, nine children (60% of the subjects, four boys and five girls) were first tested in the C condition and the remaining six (three boys and three girls) were first tested in the NC condition.

#### Games used in the experiment

We selected several common preschool games in which success depended on the ability to visually attend to the environment. Familiarity and adjustment of individual level was performed at the pretest phase. Because of the familiarity gained with each game in the pre-test phase learning effects across conditions can be excluded, though the same games were administered in the C and NC condition. See Supplementary Material for details of the games and level adjustment protocol. Children were not given a time limit to complete the games and could proceed at their own rate.

##### Puzzle assembly

Five different puzzle levels were used in the test phase, comprised of 12, 16, 20, 25, or 40 pieces, depending on the child's level, as determined in the pretest phase. The puzzle pieces were placed face-up on the horizontal board, to the right of the child. The pieces were shuffled by the experimenter, and the child was instructed to begin assembly. The child was given the same puzzle on both test-runs.

##### Lego reconstruction

**Figure 2:**
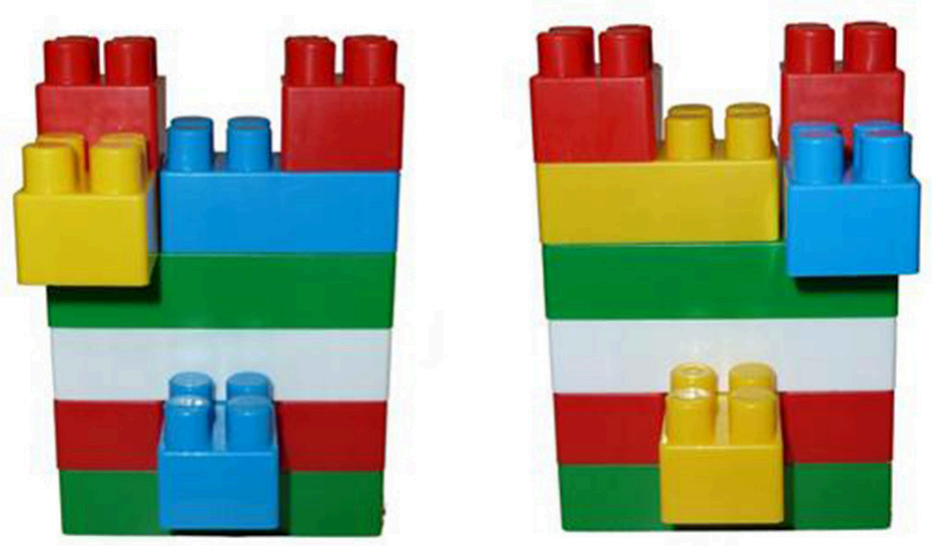
**Same-level Lego-structures**. The figure exemplifies two Lego structures of the same difficulty level that were given on different test runs.

Several large Lego blocks were placed on the horizontal surface, to the right of the child. A Lego structure (see Figure 2), which was created by the experimenter before the child entered the room, was placed on the horizontal surface in front of the child (see Figure 1), and the child was immediately instructed to duplicate it. The level of difficulty was determined during the pretest phase. The Lego game was repeated twice in each test run (i.e., the child was asked to duplicate two structures, one after the other). In both NC and C conditions, the child was given similar structures with similar color combinations, differing in minor changes, such as a mirror image, side inversion, or color inversion of one another.

##### Picture card lotto

**Figure 3:**
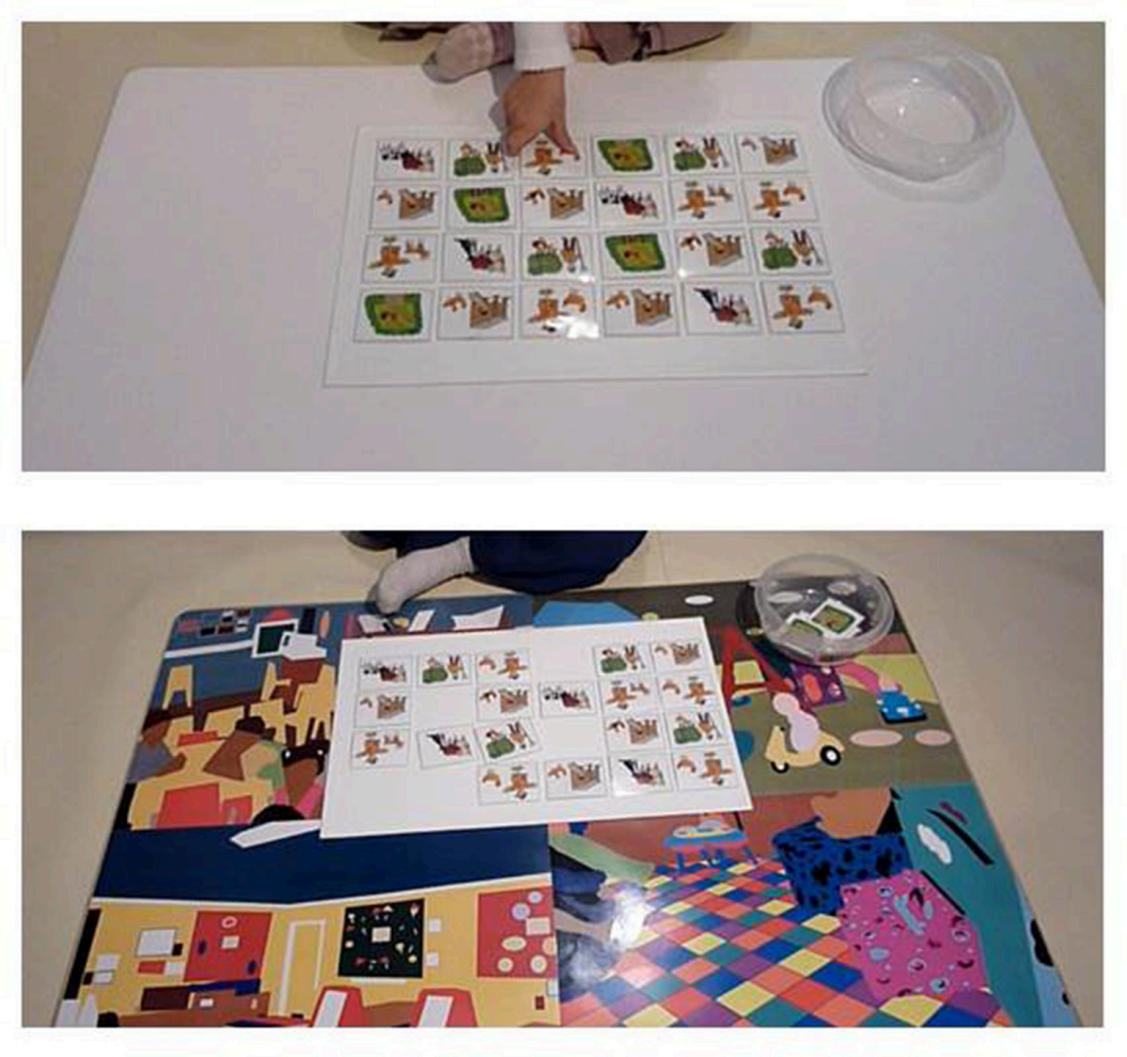
**Picture cards Lotto game**. The figure depicts the Lotto game when played on a non-colorful (NC, upper panel) or on a colorful (C, lower panel) background.

Twelve pairs of pictured cards (see Figure 3), 5.5 × 4.2 cm each, attached to a white magnetic surface (25.5 × 41 cm), were placed on the horizontal surface. The cards were arranged face up, in six rows by four columns, with the same display order for each child. The child was instructed to pick a card of his/her choice, look for its identical match, pick that card and remove it from the magnetic surface, and place the two cards in a round transparent plastic box, which was located to his/her left hand side on the horizontal surface. Selection of pairs continued until all the cards were in the box. Levels of difficulty were developed using several sets of cards in which the difference between pairs varied in saliency. Each child was given a set of cards suitable to his/her level of ability, as established during the pretest phase (see Supplementary Material). The child was given the same set of cards in both test runs.

### Test phase

#### Experimental settings

The experiment took place in a separate room in the preschool building. The room's predominant color was white, with very few colorful objects or decorations. This allowed us to control the colorfulness (in terms of contrasting hues and brightness) of the children's visual field. The light in the room was generated by a regular light bulb, placed at the center of the ceiling, and a lamp that was placed above the working surface to minimize shadows. The shutters in the room were closed during testing to minimize light condition differences. The experimenter wore the same color of clothes in both sessions for each child, and sat in the same proximity to the child on both runs. Since the normal presence of staff, children, and parents outside the room could not be restricted, we were unable to control for noise and thus regarded it as part of the ecological nature of the testing environment.

#### Audiovisual recording

Two cameras recorded the experiment. One, located above the vertical board, recorded the horizontal board and its surroundings. The second, located to the child's right, recorded his/her activities from a side view.

#### Procedure

Each child was taken individually from his/her preschool group by the experimenter (with whom s/he was already well acquainted), who informed the child that they were going to play together. The child was led to the experiment room, and was seated in front of the horizontal surface. The experimenter then engaged in a conversation with the child for at least 2 min, to allow the child to get accustomed to the room and the situation. During this time, the child was reminded of the general rules of play: s/he was to play quietly, on the horizontal surface only, and without interacting with the experimenter. The order of games was the same on all runs for all children: first puzzle assembly, then Lego reconstruction, and finally the picture cards Lotto. The game order was not randomized, whereas the order of the two conditions was randomized across subjects. Before the beginning of each game, the experimenter explained the rules of the game and asked the child to begin. As soon as the child completed the game, the surface was cleared to make room for the next game. To minimize interaction, the experimenter sat behind the child, to his/her right hand side, and avoided conversing or creating eye contact with him/her as much as possible. Children played each game twice, once in each of the two color test conditions.

### Analysis

#### Development of coding system

We analyzed the video recordings of the play sessions of the first few participants using thematic analysis (Boyatzis, 1998; Crabtree and Miller, 1999; Braun and Clarke, 2006). This method involves identifying themes in the data through a recursive process of careful watching and re-watching of the video recordings. Based on Braun and Clarke (2006), our analysis followed a process of familiarizing ourselves with the narratives of patterns of behaviors, generating initial codes, collating codes into potential themes, gathering the data relevant to each potential theme, and finally generating a thematic “map” of the analysis. The approach was to discontinue once no new patterns emerged. In total, the first nine sessions were observed. Based on this qualitative analysis, we developed a coding system, comprised of six behaviors that were hypothesized to indicate various disruptions to the execution of the task at hand (e.g., frustration, signs of tiredness, or discomfort):

- Head Approach. Head approach was defined as bringing one's head closer to the working surface more than is necessary in order to reach the games and/or its parts. For example, the child takes two pieces and attempts to connect them, while leaning forward toward the board. His elbows are bent to an angle of a little more than 90°.
- Eye Relaxation. Eye relaxation was defined as looking or turning away from the game; this movement was usually comprised of a long stare or gaze directed to a distant object. For example, the child looks upward, above the vertical board, for several seconds. He turns his head toward the experimenter, and then back to the game.
- Frustration. Frustration was defined as vocalizations or statements that indicated frustration or a feeling of incompetency (e.g., “This is hard.”). For example, the child attaches the stack of blocks to the structure, and then detaches it. A block that was in his way remains connected to the structure, and he tries to remove it, while saying “Ufff.”
- Dropping a Piece. Dropping a piece was defined an incident in which the child dropped a piece of the game (a puzzle piece, Lego block, or Lotto card) after holding it in his/her hand. For example, while trying to remove the upper piece, a group of several pieces detaches, and falls from his hands.
- Missing a Piece. Missing a piece was defined as any action taken by a child that indicated a lack of awareness of a game piece (e.g., saying that the puzzle was missing a piece when it was in front of him/her). For example, the child has two last pieces to insert into the puzzle. He stalls, looks around, turns to the experimenter several times, and then back to the board until he suddenly detects one piece and inserts it into the puzzle.
- Manual Search. Manual search was defined as a child's search for a piece with his/her hands rather than with his/her eyes. For example, the child moves her hand back and forth, groping for a piece of the puzzle.

#### Coding and reliability

A naïve coder (hourly paid) was trained to use the coding system using video segments that were excluded from the study's analysis. These video segments comprised the second Lego reconstruction task in each session (which were excluded from analysis as it seemed that in the second round children were more impatient and less motivated), and segments of a particular participant who was excluded from the analysis due to physical agitation. One of the experimenters (K.S.E) coded the same video segments and the two coders discussed disparities in their coding. This process was repeated several times until both coders felt that disparities were minimal. Then, the videos of seven randomly chosen participants that were included in the analysis were given to the two coders and the inter-coder reliability was assessed.

Inter-judge reliability for the frequency of children's interfering behaviors as assessed by intraclass correlation (ICC [2, 1]; Shrout and Fleiss, 1979) was 0.95, 0.84, and 0.94 for puzzle, Lego and Lotto, respectively. The ICC across games was 0.94. Based on the satisfactory reliabilities of the first randomly chosen seven participants' video-tapes, the experimenter continued to code the videos of the remaining eight participants and all analyses were made using only her coding scores.

#### Statistical analysis

We used paired sample *t*-tests to determine if there were significant differences between the C and NC conditions in the frequency of children's interfering behaviors. The number of occurrences of interfering behaviors in every game was normalized, to neutralize the effect of the play time. This was done using the following formula:

$$\begin{array}{l} \text{Frequency of interfering behaviors}\\ \;=\;\frac{\text{Number of occurences observed }}{\text{Time }\left[\text{sec}\right]\;}\;\times \;60 \end{array}$$

Where “Frequency of interfering behaviors” is the number of occurrences of all six behaviors combined, per second, “Number of occurrences observed” is the number of occurrences of all six behaviors combined, counted during a specific game, per a specific child, and “Time” is the number of seconds the child was engaged in the specific game (note: children were not limited at all in the time of play).

## Results

### Game duration

Since the children perceived the tasks as playtime and not as a task, as is age appropriate, we did not impose a time limit to complete the tasks; however we found no significant differences in the time it took children to complete each game in the C condition compared to the NC condition {for puzzle: mean(NC) = 5:25 min, *SD* = 3:00, mean(C) = 5:18 min, *SD* = 2:26 [*t*_(13)_ = 0.34, *p* = 0.73]; for Lego: mean(NC) = 1:41 min, *SD* = 0:39, mean(C) = 2:25 min, *SD* = 1:52 [*t*_(12)_ = −1.3, *p* = 0.21]; for Lotto: mean(NC) = 2:29 min, *SD* = 0:41, mean(C) = 2:46 min, *SD* = 0:59 [*t*_(11)_ = −1.2, *p* = 0.24]}.

### Frequencies of interfering behaviors

#### For each separate game

Table 1 depicts the frequencies of interfering behaviors for each of the three games (see first three rows). For each game, the children demonstrated between a two- to over four-fold increase in the frequency of interfering behaviors in the C condition as compared to the NC condition. This increase was significant in all games, except the Lotto. For the puzzle assembly game there was over 2.5-fold increase. For the Lego reconstruction game there was almost 4.5-fold increase. For the Lotto game there was a two-fold increase.

**Table 1:** **Frequencies of interfering behaviors**.

		**Non-Colorful**		**Colorful**		**Paired Sample *t*-test**		
	***N***	**Mean**	***SD***	**Mean**	***SD***	**df**	***t***	**Sig (2 tailed)**
Puzzle	14	0.3833	0.32704	0.9986	0.69263	13	−4.291	0.001^**^
Lego	15	0.2273	0.39631	1.0017	1.06313	14	−2.974	0.01^*^
Lotto	12	0.5092	0.45102	1.0679	1.29405	11	−1.727	0.112
Total	11	1.164	0.63995	3.4453	2.69332	10	−3.272	0.008^**^

*The table displays the mean frequencies of interfering behaviors for each of the games (first three rows), and across all games (last row) in the colorful (C) and non-colorful (NC) conditions. N is the number of children for which data was available for the particular analysis (see Section Methods)*.

* p < 0.05;

** *p < 0.01*.

#### For all games grouped together

When comparing across conditions (i.e., all interfering behaviors across all games were taken together) we found that children demonstrated a significantly higher frequency (three-fold more) of interfering behaviors in the C condition than in the NC condition (see Table 1, last row).

## Discussion

The goal of this study was to test the effect of a colorful surface background on preschool children's structured play. Children's environments are often designed to be very colorful (e.g., colored chairs, colored storage boxes, and colorful carpets. See Figure 2 in Supplementary Material for an example). Even in cases where colorfulness is not explicitly intended, the available visual view of a child playing on the floor or sitting by a table can be a cacophony of colors coming from colored toys and objects scattered nearby or the other children wearing colored clothes (see Figure 3 in Supplementary Material for an example). Accordingly, we created a proximal colorful play surface and placed it on the floor and in front of the playing child. The pre-test phase allowed us to test children having the same familiarity level with the games (children played the games after four to six repeats) and to avoid novelty effects (with the experimenter, the games, and the room), and adjust the play level to each child individually.

We hypothesized that relative to a non-colorful play surface, a colorful one would interfere with children's structured play and would induce more behaviors indicating disruption in task execution. Overall, as hypothesized, we observed a greater number of behaviors indicating disruption in task execution when children played on a colorful surface area as opposed to a non-colorful, white one. These disruption processes manifested in children behaviors, such as bringing their heads closer to the working surface, staring away, emitting vocalizations, and statements indicating frustration or feeling of incompetency, dropping, or missing pieces of the game that were in front of them, and making more use of manual, rather than visual search. Our findings are in line with previous findings with older children, indicating distracting effects of excessive proximal colorfulness (Ksantini-Hovev and Sebba, 2005) and distal colorful classroom decorations (Godwin and Fisher, 2011; Fisher et al., 2014) during various tasks.

It is interesting to speculate about the mechanisms underlying the current findings. The behaviors we observed may be due to attentional interference created by the sharp contrasts of hue and brightness at the proximal background. Indeed, previous work in the field of attention research indicates that contrasts of brightness and hue are capable of capturing attention in adults (e.g., Turatto and Galfano, 2000, 2001; Lambert et al., 2003; Fuller et al., 2009). Although, similar studies were not conducted in children, according to empirical studies in developmental psychology the ability to voluntarily control attention and avoid attention hijacking is immature in preschool children (Ruff and Capozzoli, 2003; Kannass and Colombo, 2007; Ristic and Kingstone, 2009). For example, 3- to 4-year-olds showed less abilities to voluntarily control attention than 5-year-olds (Fisher et al., 2013). Therefore, we suggest that the excess hue and brightness contrasts in the background were distracting factors, which required significant attentional resources of the children and competed for resources required for the task at hand.

Given the above *post-hoc* speculation, it is interesting to note the work of Kannass and Colombo (2007), who studied children of a similar age to the present study's participants, and with similar problem-solving tasks, in three different conditions: no distraction, intermittent (periodic) distraction, or continuous distraction. The distractors were 5-s-long segments of a TV show, which were presented either intermittently or continuously in a language foreign to the subjects. They found that the most significant impairment of attention was in the continuous condition, and suggested that at this age, children cannot tune out the constant competition for attentional focus represented by the continuous distractor, and that continuous distractors might be most disruptive for cognitive function and performance (Kannass and Colombo, 2007). Based on the present findings, we suggest that a constant but excessive colorful background can similarly serve as a continuous visual distractor competing for a child's mental resources. Indeed, some of the behaviors we identified could be interpreted as signs of attentional interference and competition. For example, head approach can be interpreted as an attempt to narrow the breadth of the attentional lens and restrict the visual area to which attentional resources are directed (an ability that is not fully developed at this age, Pasto and Burack, 1997). Eye relaxation, frustration, and dropping a piece can be interpreted as signs of depletions of these attentional resources.

Importantly, our exploratory bottom-up approach, which did not focus solely on attentional processes, enabled us to identify additional putatively disrupting behaviors that could possibly hint to further mental processes affected by excessive colorfulness. Behaviors such as manual search, missing a piece, and head approach may also be interpreted as indications of perceptual interferences. The tasks conducted by the children in our study required perceptual processes such as detection and discrimination of the game parts (puzzle and Lego pieces) from the background. Specifically, placing colorful game parts on a white background required visual detection, whereas in the colorful condition, the same task is required and in addition there is a need for activation of visual discrimination. The failure to discriminate occurs whenever stimuli are similar, and can lead to confusion (Wickens et al., 1997, pp. 102, 107). Background complexity can slow down and even hinder identification of objects (Wolfe et al., 2002). Thus, these additional processes may have contributed to the mental load and depletion created by the colorful background, and in turn, elicited behaviors indicating fatigue (such as eye relaxation, frustration, and dropping a piece).

Another potential explanation to the observed behaviors is that the novelty of the decorations rather than the visual stimulation *per se* (Imuta and Scarf, 2014) disrupted children's play behavior. Since the same children were alternated between non-colorful and colorful conditions, the transition to the colorful environment may have elicited a novelty effect. Indeed, children are known to respond strongly to novelty. Some of the most established empirical procedures to study cognitive development in children rely on children's preference to attend to novel stimuli (Imuta and Scarf, 2014). Although, in the current study children accommodated to the novel surrounding for at least 2 min before the experiment began, it may still be that some of the behaviors we observed in our study were due, at least in part, to the novelty of the colorful surface. In line with this, we suggest that future studies should assess emotional and arousal levels as an indicator for novelty effects.

The present study cannot differentiate the specific mechanisms by which excessive colorfulness may affect attentional, perceptual or other cognitive processes. However, regardless of the exact mechanisms, we suggest that the excess of hue and brightness contrasts in the colorful background demand significant mental resources (Choi et al., 2014), at the expense of the resources for normative play, leading to tiredness, fatigue, and difficulty in task execution.

Individual differences also warrant further investigation. Our findings that the colorful background induced more putative disruption-indicative behaviors are based on the average group data. However, for a few children, the colorful surface actually induced fewer of these behaviors relative to the non-colorful surface. According to several researchers, there are individual differences in arousal levels and in the way people react to and process sensory information (Dunn, 2001). While for some children the colorfulness of the surrounding may be excessive and disrupting, for others it may provide just the right amount of stimulation to support optimal arousal. Although, on average it seems that highly colorful surfaces interfere with children's play more than non-colorful surfaces, further research into the individual differences observed in this study may lead to insights into designing children's environments in ways that can be helpful for individuals with different types of sensory and arousal profiles.

As mentioned in the previous section, findings regarding disruptive behaviors were obtained for all the games taken together. A closer look revealed that these differences were more pronounced in the puzzle and Lego games, while the difference for the Lotto task did not reach significance. As the Lotto part was always the last game, this finding may stem from an accumulated effect of tiredness and mental depletion. Another explanation is that the white magnetic board underneath the picture cards in the Lotto game (see Figure 3) helped reduce the distracting effects of the colorful background and thus reduced the difference between the two conditions. This suggests that it may be helpful to delimit the working space of children with a color-quiet surrounding to attenuate effects of colorfulness in the periphery of the visual field. Further research is required to assess whether indeed an immediate neutral background can buffer effects of the colorful surroundings.

There are several limitations to our study and conclusions. A major limitation of this pilot study is the small sample size of 15 subjects. This prevented randomization of the order of the games. This sample size also did not allow a thorough analysis of inter-subject differences. Second, although running the experiment in the preschool setting had many advantages, the downside of this approach was the difficulty to control for all variables. For example, there was no control over the colorfulness of the children's clothing and accessories. Thirdly, though one of the coders was naïve as to the purpose of the experiment, the investigator that played with the children and was involved in the development of the coding scheme (K.S.E), was not blind to the hypothesis of the experiment. Fourthly, children were not screened for color blindness. Although we assume that the brightness in contrasts would be visible to colorblind people, and if any, colorblindness in one or more individuals would strengthen our results, we have not directly tested this point. Fifthly, our setup enabled us to study disruption to the structured play but not the direct effects on task performance. Children were not given a time limit nor directed to explicitly announce when they were finished. Future studies should design age-appropriate tasks in which success can be measured more directly. Finally, our study focused on situations in which the play area (as designed intentionally or as perceived from a child's viewpoint) was highly *colorful*. Importantly, we did not study the effect of colored surfaces composed of *singl*e colors. Our results cannot be generalized to play environments that use less variety of colors, or when children engage in structured play with only one color as a background.

In summary, the present findings suggest that excessive colorfulness, which is often used to create enriched environments and is considered developmentally beneficial to children, impacts children's behavior in ways that may be indicative of interference to their engagement in structured play tasks. Based on our results, and based on previous findings in older children (Ksantini-Hovev and Sebba, 2005; Godwin and Fisher, 2011; Fisher et al., 2014), we suggest that caution is warranted when designing colorful children's environments. Further studies should elaborate this investigation, extending it to other sources of stimulation (e.g., noise, light, smell etc.), different play environments, and the potential of moderating effects.

## Author contributions

RS, NL, and KS designed the experiment and performed the analysis; RS and KS developed the coding system; KS ran the experiment and preformed the analysis; SZ helped with the qualitative analysis; KS and NL wrote the manuscript.

### Conflict of interest statement

The authors declare that the research was conducted in the absence of any commercial or financial relationships that could be construed as a potential conflict of interest.
