# Supplementary material for: Disruptive Effects of Colorful vs. Non-colorful Play Area on Structured Play—A Pilot Study with Preschoolers
Source: Front Psychol. 2016 Oct 28;7:1661. doi: 10.3389/fpsyg.2016.01661 (PMC5083879; doi:10.3389/fpsyg.2016.01661)
Supplement: Supplementary file 1 [file DataSheet1.pdf]

Supplementary Material

Disruptive Effects of a Colorful versus Non-Colorful Play Area on the Performance of Preschoolers during Structured Play – a Pilot Study

Keren Stern-Ellran[1], Sigal Zilcha-Mano[2], Rachel Sebba[1], Nava Levit-Binnun[3,*]

[1]Faculty of Architecture and Town Planning, Technion – Israel Institute of Technology, Haifa, Israel

[2]Department of Psychology, University of Haifa, Haifa, Israel

[3]Sagol Center for Brain and Mind, Baruch Ivcher School of Psychology, Interdisciplinary Center (IDC) Herzliya, Herzliya, Israel

*** Correspondence:**
Corresponding Author
navalb@idc.ac.il

1.1 Pre-Test Phase

The work at the preschool commenced with a long pre-test phase. The goal of the pretest phase was to adjust the level of each of the games for each and every child. In addition, the pretest phase served as a means to minimize any effects of unfamiliarity with the games, with the experiment room or the experimenter. To this end, the experimenter visited the preschool on a regular basis, and played with every child individually inside the preschool's space across several occasions that spanned several weeks.

1.2 Accommodation with the experiment

In the first part of the pre-test phase, the experimenter played with every child individually inside the preschool's space, in eye contact with the other children and staff. At this stage, the emphasis was placed on providing the child with a positive experience, and on becoming acquainted with the experimenter.
In the second part of the pre-test phase, the play area was moved to a separate room, next to the preschool's main room where even behind the closed door one could still hear the ongoing activity in the preschool. Every once in a while, children and/or staff entered the room for a short time.

The last part of the pre-test phase included rather intensive preparation, in which we repeated the tasks to be used in the test phase several times with every child. In order to help the children become familiarized with the test phase setting, the last session of the pre-test phase was conducted in the experiment room, exactly according to the non-colorful (NC) condition of the test-phase protocol, but no data were recorded.

1.3 Adjusting game difficulty

The games used for the test are described in the main article. During the pre-test phase we realized that despite the relatively small age differences between the children, there were significant differences in their capabilities. Due to these differences between children, several levels of complexity were created for each task and the appropriate level in each task was determined for each child (see for example Figure 1 for the Lego game). The goal was to find a level that is challenging (i.e., not too easy, and not too difficult). We assumed that if a task were too easy, one could complete it almost regardless of interruptions, and when too difficult, even the most supportive environment would not promote success. Thus, for each child, we found a moderately challenging level which was neither too easy nor too difficult. In this "possible challenge range", we assumed that a supportive environment might encourage success.

1.3.1 Puzzle assembly

Different puzzles sizes were used and optimal puzzle size was determined for each child. For example, a child was given a 12-piece puzzle. If she completed the puzzle easily, then she was given a 16-piece puzzle, and so on, until she could finish the puzzle with little or no help, but needed to pause several times to think, and made several attempts to attach a piece to a wrong position.

1.3.2 Lego reconstruction

In the Lego game, children were asked to duplicate a Lego structure, which was created by the experimenter before the child entered the room (see main article for details).

Different levels of difficulty were created by varying the number of Lego tiers, and the structural features (see examples in Figures 1). For each child, the level of difficulty was increased so long as the child was able to replicate the structure easily and with little to no assistance. Level was set when the child seemed challenged (e.g., required several attempts, paused to think and plan, reevaluated her performance, in order to achieve satisfactory –perfect or nearly perfect – resemblance to the original structure).

1.3.3 Picture cards Lotto

The Lotto game was described in detail in the main article. Levels of difficulty for the Lotto game were developed using several sets of cards in which the saliency of the differences within pairs varied. For example, in the easy set of cards, each pair displayed a different animal, while in the more challenging set of cards, the same animal was displayed in several pairs, but there were subtle differences in color or number.

2. Creation of the colorful background

The rational for the creation of the background was taken from Sebba, 2005. She and others (e.g., Smith et al., 2015) emphasize the importance of considering the visual environment from the child's point of view in order to understand various developmental processes (Smith et al., 2011). Indeed, when pictures of children's play environments are taken from the child's perspective (playing on the floor or next to a table, or sitting inside a playground facility), the available visual environment that emerges is often more colorful then when perceived by a standing adult viewing the room from above or the playground from afar (Sebba, 2005; see Figure 3 for examples). Such excessive colorfulness may occur, for example, when children play on colorful carpets (e.g., see Figure 2c or google “children’s’ carpets”), or surrounded by colorful toys, cups or chairs, or other children wearing colorful clothes (Figure 3).

To simulate such situations, the colorful background was created using Photoshop (Adobe Systems Software Ltd) processing of pictures taken of children's play environments (see Figure 2, right column). The purpose of the processing was to make objects and details less identifiable, while enhancing the hue and brightness contrasts. In addition, the pictures were placed upside-down, to further reduce the chance of object perception, which may distract the child from the game. All children were tested on the same colorful background in the colorful condition (see Figure 1 in main manuscript).

Figures:

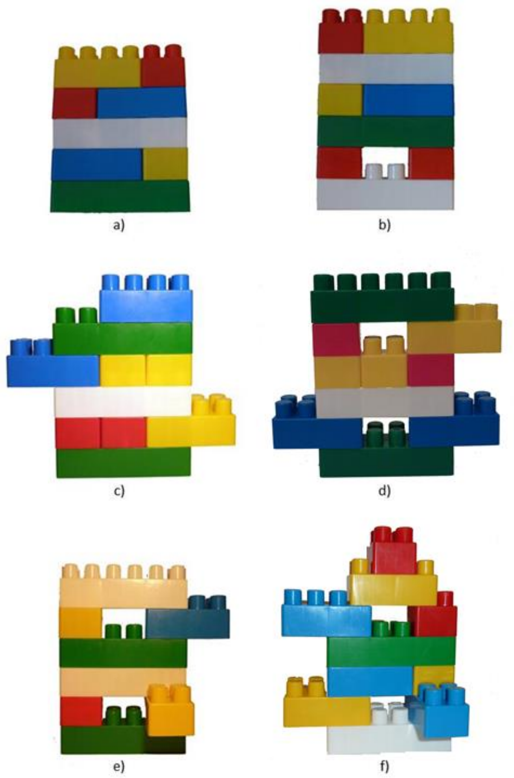

Figure 1: Examples of Lego structures varying in level of diffculty. Despite the relatively small age differences between the children, there were significant differences in their capabilities. Thus, several levels of complexity were created for each task and the appropriate level in each task was determined for each child. The picture displays different levels of complexity for the Lego task. The upper-most panel is an easier level and the lower-most panel is a more difficult level.

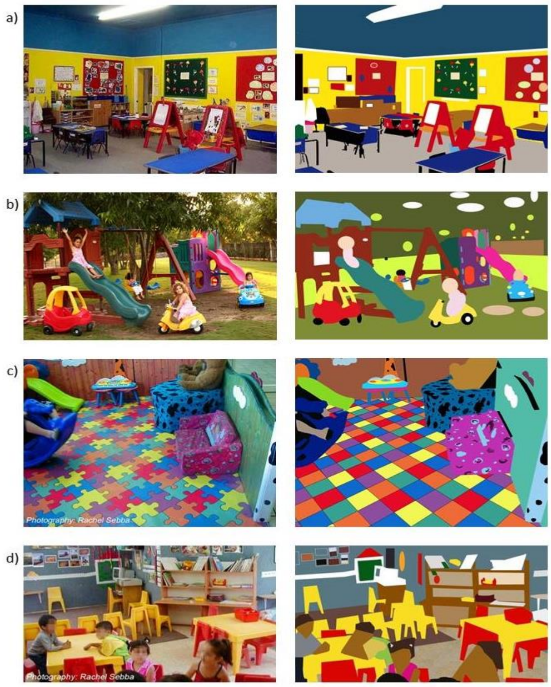

Figure 2: Examples of children's colorful play environemnts and the colorful backgrounds created from these pictures for the study. On the left are the pictures that were used to create the colorful background. These are all pictures of colorful children's enviroments, often comprised of saturated colors, and contrasts of brightness and hue. On the right are these pictures after Photoshop processing. The processed pictures were connected together and placed upside down, to minimize object perception.

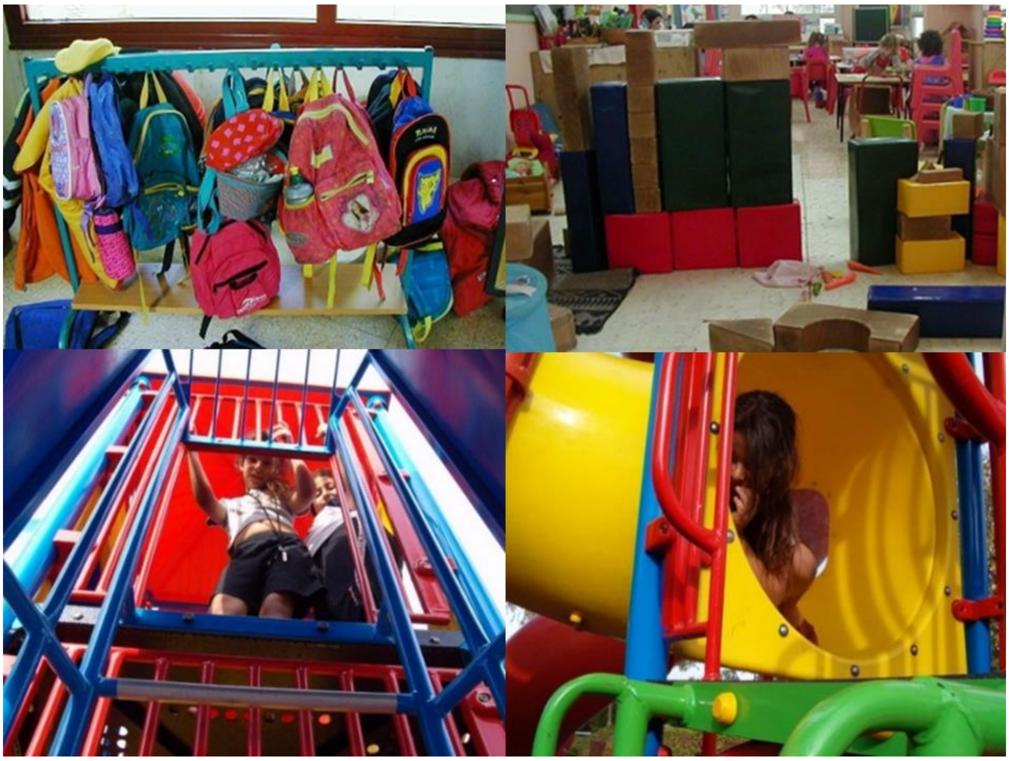

Figure 3: Pictures of play environments taken from the child's perspective. Even areas that are comprised of large colored surfaces (one color and not many; e.g., playground facilities), or areas that were not intended to be colorful (e.g., clothes rack), can give rise to a cacophony of colors from some viewpoints.

References

Sebba, R. (2005). Color in Educational Buildings - Theory and Practice. Jerusalem: Ministry of Education and Culture.

Smith, L. B., Yu, C., & Pereira, A. F. (2011). Not your mother's view: The dynamics of toddler visual experience. Developmental Science, 14(1), 9-17

Smith, L. B., Yu, C., Yoshida, H., & Fausey, C. M. (2015). Contributions of head-mounted cameras to studying the visual environments of infants and young children. Journal of Cognition and Development, 16(3), 407-419.
